# *Ex vivo* induction of antitumor DEC-205^+^ CD11c^+^ cells in a murine neuroblastoma model by co-stimulation with doxorubicin, lipopolysaccharide and interleukin-4

**DOI:** 10.3892/br.2020.1351

**Published:** 2020-08-27

**Authors:** Seiichiro Inoue, Yumiko Setoyama, Yoshifumi Beck, Daiki Kitagawa, Akio Odaka

Biomed Rep 4:27–32, 2016; DOI: 10.3892/br.2015.546

After the publication of the above paper, the authors have realized that the address associated with the second affiliation was presented incorrectly: “Department of Medical Research” should have been written as “Department of Biomedical Sciences”. Therefore, the authors’ names and affiliations in this paper should have been presented as follows:

SEIICHIRO INOUE^1^, YUMIKO SETOYAMA^2^, YOSHIFUMI BECK^1^, DAIKI KITAGAWA^1^ and AKIO ODAKA^1^

Departments of ^1^Hepato-Biliary-Pancreatic and Pediatric Surgery and ^2^**Biomedical Sciences**, Saitama Medical Center, Saitama Medical University, Kawagoe, Saitama 3508550, Japan

The authors regret that this error with the author affiliations was not noticed prior to the publication of their paper, and apologize for any inconvenience caused.

